# Familial Blue Rubber Bleb Nevus Syndrome in Pregnancy with Spinal Epidural Involvement

**DOI:** 10.1155/2013/141506

**Published:** 2013-05-09

**Authors:** Daigo Ochiai, Kei Miyakoshi, Kazumi Yakubo, Tatsuro Fukuiya, Yasunori Yoshimura

**Affiliations:** ^1^Department of Obstetrics & Gynecology, Saitama City Hospital, 2460 Mimuro, Midori-ku, Saitama-shi, Saitama 336-8522, Japan; ^2^Department of Obstetrics & Gynecology, School of Medicine, Keio University, 35 Shinanomachi, Shinjuku-ku, Tokyo 160-8582, Japan

## Abstract

Blue rubber bleb nevus syndrome (BRBNS) is a rare vascular disorder characterized by multiple venous malformations (VMs) of the skin, gastrointestinal tract, and other organs. To date, several cases of sporadic BRBNS involving various parts of the pregnant woman's body have been reported; however, BRBNS in pregnancy with spinal epidural involvement has not been reported. Here, we describe the clinical features and management of familial BRBNS in pregnancy. The patient presented with multiple VMs on her head, neck, floor of the mouth, trunk, leg, foot, and vulva and spinal epidural lesions. The patient's mother and sister also exhibited multiple VMs similar lesions, indicating a familial form of BRBNS. Cesarean section under general anesthesia was performed, and a healthy male neonate was delivered. The mother's postoperative course was uneventful and her VMs decreased in size after delivery. Physicians should consider the possibility of systemic diseases and familial inheritance in cases of VMs.

## 1. Introduction

Blue rubber bleb nevus syndrome (BRBNS) is a rare vascular disorder characterized by multiple venous malformations (VMs) of the skin, gastrointestinal tract, and other organs. BRBNS is typically sporadic, and familial cases have been rarely reported [1–4]. To date, several cases of sporadic BRBNS in pregnancy have been reported, with involvement of the pregnant woman's neck, floor of the mouth, trunk, leg, foot, vulva, and placenta [5–9]. However, BRBNS in pregnancy with spinal epidural involvement has not been reported. We describe the clinical features and management of familial BRBNS in pregnancy.

## 2. Case Report

**Figure 1:**
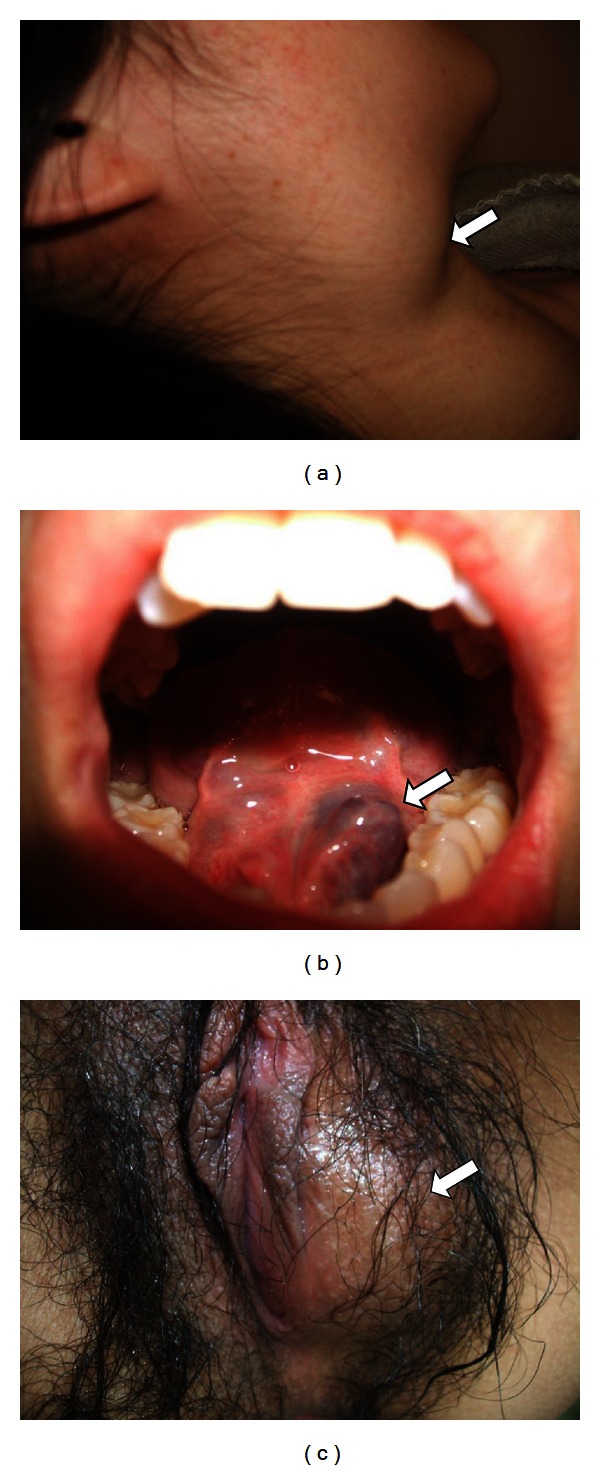
Venous malformations in pregnancy. Multiple venous malformations were observed on the patient's (a) neck (arrow), (b) floor of the mouth (arrow), and (c) vulva (arrow).

**Figure 2:**
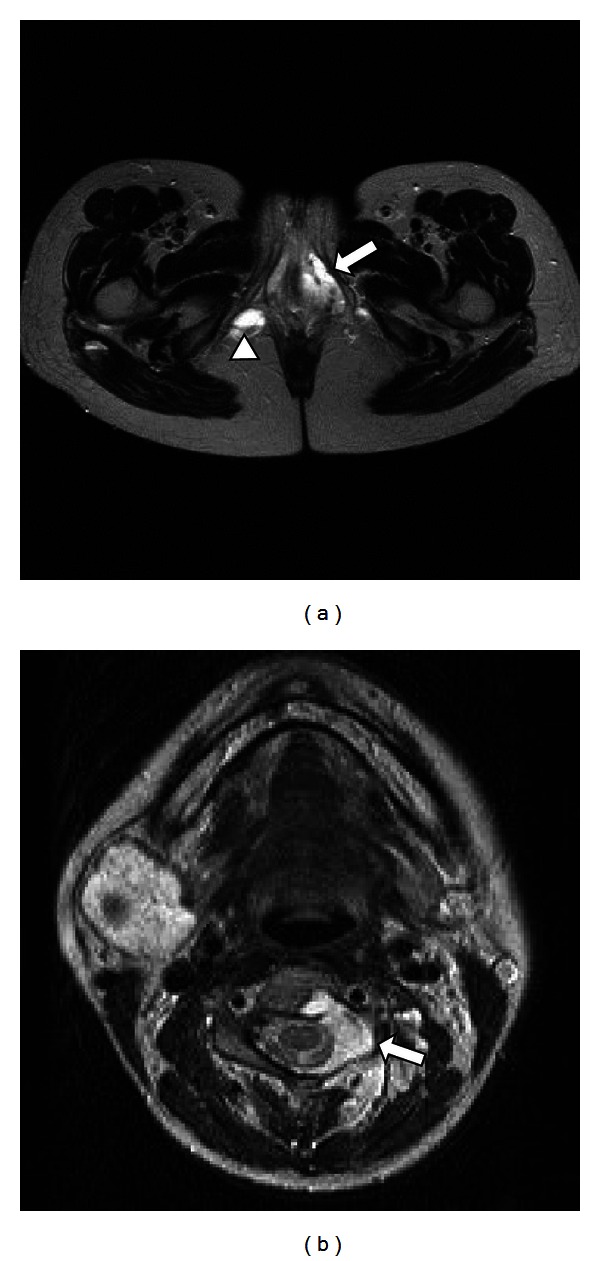
T2-weighted pelvic and brain magnetic resonance imaging (MRI). (a) Pelvic MRI demonstrating venous malformations in the left vulva (arrow) and right levator ani (arrow head). (b) Brain MRI exhibiting spinal epidural venous malformations (arrow).

 A 36-year-old nulliparous woman was referred to out hospital because of preterm labor at 33 weeks of gestation. On admission, we observed multiple, soft, compressible, and painless lesions of VMs on her head, neck, floor of the mouth, trunk, leg, foot, and vulva (Figure 1). At the age of 25 years, she was diagnosed with multiple VMs on the basis of histological features. An endoscopic examination performed at that time demonstrated no lesions in her gastrointestinal tract. Her mother and sister also exhibited multiple VMs, indicating familial BRBNS. Pelvic magnetic resonance imaging (MRI) performed at 35 weeks of gestation to assess the birth canal revealed VMs in the right levator ani and left vulva (Figure 2(a)). Although a neurological examination revealed no deficits, T2-weighted brain MRI revealed a hyperintense VM within the spinal epidural canal at the C3 vertebral level (Figure 2(b)). However, VMs were not apparent in the upper airway, and her pregnancy course was uneventful. To avoid uncontrollable bleeding from the birth canal, cesarean section under general anesthesia was performed at 36^5/7^ weeks of gestation because of labor pain. A 3225 g male neonate with Apgar scores of 6 and 8 at 1 and 5 min, respectively, was delivered. The mother's postoperative course was uneventful, and her VMs decreased in size after delivery. Now, the child shows no evidence of VMs for 3 years. However, long-term follow-up is recommended.

## 3. Discussion

 The diagnostic criteria for BRBNS include a clinical evaluation of the cutaneous lesions. Small, multifocal, cutaneous, and/or mucosal, bluish-purple vascular lesions are usually present at birth; new lesions may appear over time [1–9]. BRBNS usually occurs sporadically but may also be inherited as an autosomal dominant or sex-linked disorder [1–4]. In this case, the clinical features of the patient were consistent with those of her family members, leading to a clinical diagnosis of familial BRBNS. Although the patient's son showed no evidence of VMs during the neonatal period, he should be followed up for a longer period of time to monitor the development of VMs. 

 VMs can increase in size and sporadically develop during pregnancy, as observed in our patient with lesions of the spine and birth canal (i.e., the right levator ani and vulva). The physiological, vascular, hemodynamic, and hormonal changes in pregnancy increase the size of the preexisting VMs; most of these changes peak during the third trimester of pregnancy [10]. The most important contributing factor is the increase in venous pressure resulting from mechanical obstruction of blood flow by the gravid uterus [10]. In our case, brain MRI performed to confirm VM involvement of the upper airway revealed a spinal epidural lesion that developed during pregnancy, although the patient was neurologically asymptomatic. Several authors have reported symptomatic cases of spinal epidural VMs in pregnancy, for which the patients underwent laminectomy and embolization during the perinatal or postpartum period [10–12]. In contrast, asymptomatic cases of spinal lesions should be followed up carefully during pregnancy and the postpartum period [10].

 VMs in close proximity to the birth canal are considered a contraindication to vaginal delivery because they are associated with a high risk of uncontrollable bleeding [6, 7, 9]; therefore, elective cesarean section was performed in this case. Although regional anesthesia is usually employed for cesarean sections, a standard anesthetic method for systemic VMs, such as BRBNS and Von Hippel-Lindau disease, remains controversial, particularly in cases of nervous system involvement [13–15]. General anesthesia was selected because of the following reasons: MRI revealed no VMs in the patient's air way, direct visual guidance during the procedure is preferred, and spinal or epidural anesthesia may lead to secondary spinal epidural hematoma induced by direct or indirect injury to the vasculature [16]. However, further studies are needed to confirm the advantages of general anesthesia.

 In conclusion, physicians should consider the possibility of systemic diseases and familial inheritance in cases of VMs. MRI for systemic assessment should be performed as near to delivery as possible because pregnancy may cause existing VMs to worsen or may induce the formation of new lesions.
